# Reinforced Concrete Structure Performance in Marine Structures: Analyzing Durability Indexes to Obtain More Accurate Corrosion Initiation Time Predictions

**DOI:** 10.3390/ma14247662

**Published:** 2021-12-12

**Authors:** Mauricio Arreola-Sanchez, Elia M. Alonso-Guzman, Wilfrido Martinez-Molina, Andres A. Torres-Acosta, Hugo L. Chavez-Garcia, Jose M. Ponce-Ortega

**Affiliations:** 1 Faculty of Civil Engineering, Universidad Michoacana de San Nicolas de Hidalgo, Morelia 58060, Mexico; mauricio.arreola@umich.mx (M.A.-S.); wilfrido.martinez@umich.mx (W.M.-M.); luis.chavez@umich.mx (H.L.C.-G.); 2 Tecnológico de Monterrey, School of Engineering and Sciences, Querétaro Campus, Querétaro 76130, Mexico; atorresa@tec.mx; 3 Faculty of Chemical Engineering, Universidad Michoacana de San Nicolas de Hidalgo, Morelia 58060, Mexico; jose.ponce@umich.mx

**Keywords:** reinforced concrete structures, service life, data cleaning, electrical resistivity

## Abstract

This paper presents a comparison of six index properties collected during durability inspections of five Mexican seaports. Typical durability indicators such as compressive strength, saturated electrical resistivity, ultrasonic pulse velocity, percent total void content, capillary porosity, and chloride concentration profiles were analyzed to obtain empirical correlations with the non-steady-state chloride diffusion coefficient. These indices were compared to determine correlation coefficients that are the most important for obtaining better corrosion initiation forecasting. Two models of corrosion initiation time (t_i_) were used: Fick’s second law of diffusion and the reported UNE-83994-2 (Spanish Association for Standardization, UNE) in which electrical resistivity was used to calculate concrete service life. The data from both models were cleaned using correlated variables, and the initial variables were compared with t_i_. The main result achieved was the verification of the feasibility of using correlations of variables to clean unnecessary data in order to calculate t_i_. Additionally, electrical resistivity was identified as one of the main durability indexes for in-service concrete structures exposed to marine environments. This is important because electrical resistivity is a non-destructive and reliable test that can be measured both in the laboratory and in the field very easily.

## 1. Introduction

The most widely used material in construction is concrete. The durability of concrete has gained great relevance in the last decades. Hence, a large number of studies have explored the subject, including the analysis of reinforcing steel corrosion (in marine and urban/industrial environments), alkali-aggregate reactivity, sulfate attack, freezing and thawing [1].

Deterioration is present in all concrete structures regardless of the quality of their design and construction. This phenomenon has become evident and worrisome for many modern structures (in marine environments) that present strong and premature deterioration, along with low durability [2]. This condition of modern structures may be due to the design criteria for durability being stricter than the design criteria for structural resistance, thereby implying overdesign for structural engineers and additional costs [3].

The main mechanism of damage to a reinforced concrete (RC) structure is corrosion, especially in marine environments, and this process impairs structural safety by decreasing the load capacity of structures [4,5,6]. In RC structures, corrosion can be divided into two stages: (i) corrosion initiation and (ii) corrosion propagation. In stage (i), chloride ions (chlorides) penetrate concrete (and/or carbonation, which is out of the scope of this paper) while steel remains passivated [7,8]. Once the reinforcing steel is depassivated, the corrosion propagation process begins and thus implies a continuous loss of an effective cross section of the reinforcing steel. Therefore, increasingly important undertakings include the design of durable structures, prediction of the remaining useful life of existing structures and development of adequate strategies to extend their useful life. In the last three decades, these requirements have become the focus of several studies that aim to model the ingress of chlorides through concrete and to estimate the initiation and propagation times of corrosion [9,10,11,12,13].

Depending on environmental exposure conditions and moisture content of concrete, aggressive agents such as chlorides realize infiltration by using different combinations of transport mechanisms. In addition to chlorides, water and oxygen (necessary for corrosion) penetrate concrete through pores, paste/aggregate interface and microcracks present in the concrete matrix [14].

Several durability tests have been performed to determine the level of attack and aggressiveness of many types of aggressive environments. Permeability, i.e., the resistance of concrete to fluid penetration, is the main property that should be minimized to ensure adequate protection against any type of exposure [15,16].

Permeability is the ability of a material to allow or limit the passage of fluids and gases through it under a pressure gradient and is affected by different parameters [17]. Several studies have tried to identify the correlation between this property and the different parameters that affect it to decrease the level of incidence [18,19,20,21,22,23,24]. The permeability of concrete decreases rapidly during cement hydration because of the increase in the formation of cement hydrates, which results in the pores originally filled with water being occupied by these hydrates [20,21,22,23,24]. The effects of curing conditions, types of constituent materials and water-to-cement ratio (w/c) by weight on permeability have also been investigated [25,26,27]. Several studies have shown that permeability increases with high w/c ratios because of increased porosity. Results have indicated that the w/c ratio particularly influences capillary porosity, pore systems and permeability [27,28,29,30].

Most deterioration processes consist of two stages. In the first stage, the deterioration promoting agents (water, ionic solutions with dissolved salts and gases) need to be transported through the capillary pores of concrete toward the reaction zones (e.g., chlorides corroding the reinforcement or sulfates reacting with aluminates) before actual chemical or physical deterioration reactions [31].

One of the main causes of corrosion of RC is the presence of chlorides in reinforcing steel. Chlorides affect the service life of structures exposed to this type of aggressive environment. Depending on the particular environmental conditions at each construction site, a single mechanism or several diffusion mechanisms acting in combination may transport chlorides inside concrete [31,32]. In the case of the submerged parts of marine structures in which concrete is saturated with water, the mechanism that dominates the entry of chlorides seems to be diffusion [33]; as depth increases, chloride content decreases. However, when concrete is subjected to wet–dry cycles, as in the case of tidal zones or environments with the presence of deicing salts, it is regarded to be in an unsaturated state [34]. Under these conditions, diffusion and capillary suction drive the migration of chlorides into the concrete in a combined manner [35]. The phenomenon of chloride transport in wet–dry cycles has been extensively studied. A maximum chloride content has also been set, with the amount decreasing as the depth relative to the exposed surface of the concrete increases [9,36,37,38,39].

Penetrated chlorides generally exist in two main forms within concrete: bound chlorides and free chlorides. Of these two forms, only free chlorides are directly detrimental to the risk of reinforcement corrosion, but some bound chlorides may be released at later stages and added to the free chlorides [40,41,42,43,44]. As a result of the binding of chlorides to some internal components of concrete, the time required for free chlorides to reach a threshold level (and trigger corrosion) increases considerably [45,46]. Chloride ions from the external environment migrate through microfractures or pores within the concrete cover until they reach a concentration that is sufficient for the passivation layer of the embedded steel to be destroyed. The condition is conducive to the initiation of steel corrosion. This phase can be described by Fick’s second law [47].

Mexico has 16 federal international ports, and all of them have a strong impact on the country’s economy and require strong investments for their maintenance and expansion. In 2006, the impact of the port maritime subsector on the national gross domestic product amounted to 1.6 billion USD and generated around 157,000 jobs [48]. The main problems for ports’ infrastructure are the adverse environmental conditions, with ports being in direct contact with seawater or brackish water, and their inadequate use by port operators (e.g., overloading the structures); these problems directly impact the performance of the structures and significantly decrease their service life. Hence, periodic interventions involving the reintegration of the operability of such structures are necessary [49].

Several researchers have investigated the durability of concrete, and the results have broadened our understanding of underlying phenomena and have resulted in improved specifications and design for durability. However, our understanding and application of such results to real structures are still limited. Recently, the Nanocem Consortium (Nanocem Workshop: Deterioration Mechanisms. Portmarnock, Ireland, November 2018) highlighted the need to advance the understanding of key deterioration mechanisms, especially in applications under real construction situations [2]; this topic is the focus of the present study.

The present study is focused on obtaining the correlation coefficients between the most important durability indices (compressive strength, saturated electrical resistivity, ultrasonic pulse velocity, percent total void content and capillary porosity) and non-steady-state chloride diffusion estimates (calculated from acquired chloride concentration profiles) based on data gathered from several marine natural exposure sites. The correlation coefficients between these durability indices were analyzed to determine the impact of all these indices in order to validate their individual importance on corrosion initiation predictions in a marine environment.

## 2. Materials and Methods

### 2.1. Study Description

**Figure 1:**
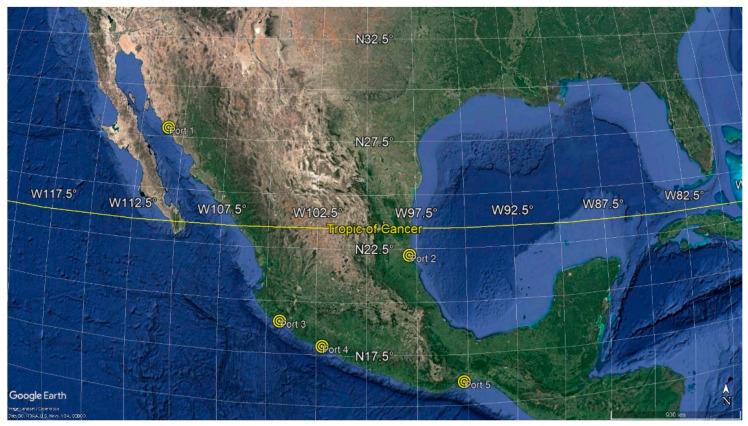
Location of the five Mexican seaports studied.

This study analyzes data collected during the durability inspection of 47 reinforced concrete piers (typical dimensions 25 m × 250 m) located on five Mexican seaports. These inspections were carried out at the request of Mexico’s Secretariat of Communications and Transportation (SCT), choosing these ports based on their commercial, historical and strategic importance. One seaport is located in the Gulf of Mexico while the other four are located on the Pacific coast. The naming of ports is related to their north to south positions; thus, port 1 is found in the farthest north position, and port 5 is found in the farthest south position (Figure 1). All the evaluated reinforced concrete piers were constructed between the 1970s and the 1980s, and all presented different construction systems, different construction Standards and material´s specifications used and different aggregate and Portland cement types. The durability inspections included concrete core extractions on different structural elements at each pier evaluated: slabs, pile caps, piles and facades. All extracted cores were then selected for laboratory testing in order to obtain the durability indices listed before. The main contribution of this study is precisely that, in the face of so much variation in concrete characteristics, it has been possible to determine a suitable chloride diffusion coefficient involving six index properties, with ER being the property that best validates the prediction models. Table 1 shows the analyzed ports.

### 2.2. Studied Variables

The data obtained during our inspections consist of the levels of damage of the different structural elements and the details of the physical, mechanical, chemical and electrochemical tests on such elements. With these data, we aim to provide a diagnosis of the status of each port enclosure and to assess the urgency of the need for repair based on the results obtained from the characterization of concrete and the level and incidence of observed damage. In this study, the following characteristics of concrete that make up the different berthing structures of the port enclosures are considered (these characteristics are presented in the “Manual for the inspection, evaluation and diagnosis of corrosion in RC structures” of Thematic network “DURAR” [50]): compressive strength (fc), non-steady state diffusion coefficient of chloride ion (D_nss_), effective porosity (EP), total porosity (TP), electrical resistivity (ER) and ultrasonic pulse velocity (UPV). Comparisons are made between the different characteristics measured and analyzed during field inspections to determine which of them are correlated.

Preventive maintenance can be performed before deterioration spreads if adequate information, modeling and planning are available. Such process maintains the serviceability of buildings and even extends their service life [51]. At present, the life cycle of buildings can be improved, the service life of structures can be increased and the life cycle cost and environmental impact of structures can be minimized by applying optimization algorithms in planning maintenance activities [52,53,54,55,56].

### 2.3. Models of the Concrete Deterioration Process

**Figure 2:**
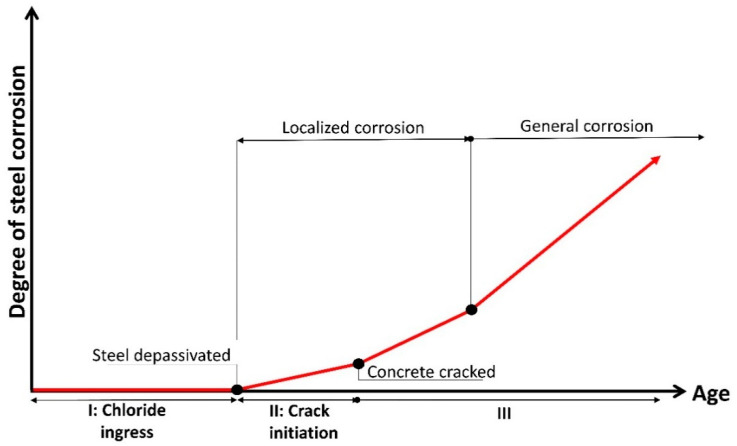
Schematic of three-phase deterioration of RC structures.

The most common method to model concrete deterioration caused by chlorides is to adopt a three-phase process, which involves chloride ingress, crack initiation and crack propagation [57,58] (Figure 2). The first phase is the time from the completion of the construction of the structure to the corrosion initiation. This first phase has been investigated within the area of reinforcement corrosion in the last decades [59,60,61].

This first phase is called the “initiation time (t_i_)” in the reported Resistivity-based model [62,63], which describes it as the total life of RC relative to the corrosion of the reinforcement, that is, the sum of the period until the reinforcement corrodes or the initiation period (t_i_) in addition to the propagation period of corrosion (t_p_). In the current study, it is used only to determine the initiation period, and it is calculated with two different models.

#### 2.3.1. Fick’s Second Law

Chloride ingress can be described by Fick’s second law [64]. Under the assumption of time-invariant surface chloride concentration (C_s_: mass percentage of binders) and diffusion coefficient (D_nss_: mm^2^/s), the chloride concentration (C_x_: mass percentage of binders) at a certain depth (x: mm), an estimated initial chloride content (C_i_: mass percentage of binders) and a certain time (t_i_: s) can be expressed as follows:

C_x_ = C_i_ + (C_s_ − C_i_)·(1 − erf [x/2(D_nss_ × t_i_)^1/2^]) (1)

where erf is the mathematical error function. In order to deal with the fact that the chloride diffusion coefficient is time-variant [65], this study, in addition to C_s_ and C_i_, adopts a non-steady-state diffusion coefficient in the model, the procedure of which is described in standard NMX-C-546-ONNCCE-2018.

In order to estimate initiation time (t_i_), this study uses Equation (2) and takes the chloride threshold value (C_x_) as indicated in the NMX-C-523-ONCCE-2016 standard.

t_i_ = (x^2^/4D_nss_) × erf^−2^ × [(C_x_ − C_i_)/(C_s_ − C_i_)] (2)

#### 2.3.2. Calculation of Useful Life Concerning the Corrosion of Reinforcements through Resistivity (Resistivity-Based Model)

The Resistivity-based model [62,63] allows the calculation of the life of a structure concerning rebar corrosion during initiation and propagation periods; in this calculation, the ER of concrete is used as a controlling parameter. This method is newly developed; although no method is calibrated, it is less widespread than the one based on Fick’s second law.

The method is based on the relationship between diffusivity and resistance to the movement of electrical loads (ions) established about 100 years ago by A. Einstein. It has been modified by other authors in order to apply the principle to porous media. This relationship is expressed as follows [63]:

D_e_ = k_cl_/ρ_es_ = k_cl_ σ (3)

where D_e_ is the effective diffusion coefficient (it does not consider the reaction of chlorides or carbon dioxide with the cement phases), k_cl_ is a constant that depends on the outer concentration of chlorides, ρ_es_ is the resistivity of saturated concrete (in this case, it is the value at 28 days of wet curing, similar to mechanical resistance) and σ is the conductivity (inverse of resistivity). In water-saturated concrete, resistivity indicates the level of porosity (pore connectivity) and is, thus, directly related to the transportation phenomena of aggressive agents (chlorides, sulfates, etc.).

However, this basic equation is insufficient for modelling the case of concrete life as chlorides are combined with the cement phases and the properties of concrete evolve. Therefore, the following concepts are introduced.

Delay factor (r_Cl_) is introduced to take into account the amount of chlorides that do not advance because they combine with the cement phases. For this purpose, Einstein’s relation has been modified by establishing an apparent resistivity as follows [62,63]:

D_ap_ = F_Cl_/ρ_ef_ × r_Cl_ = F_Cl_/ρ_ap_ (4)

where

D_ap_: Diffusion coefficient in non-steady state referred to cm^3^ of the sample in cm^2^/s obtained according to UNE 83,986 standard;
F_Cl_: Environmental exposure factor that takes into account the degree of environmental aggressiveness and chloride content;
ρ_ap_: Apparent resistivity. It is the value of the effective resistivity multiplied by the reaction factor (r_cl_).

Age factor (q) represents the resistivity evolution with time. This factor may not be used if accelerated curing is performed or if the structure is more than one year old.

The new equation (Equation (4)) is, thus, written as follows [62,63,66]:

t_i_ = [(x^2^ × ρ_ef,0_ × (t_n_/t_0_)^q^)/F_Cl_] × r_Cl_ (5)

where

x: Minimum concrete cover thickness, cm;
ρ_ef,0_: Effective resistivity at 28 days of curing, Ω cm;
q: Age factor, (-);
t_0_: First age of 28 days in which the value of resistivity is taken;
t_n_: Latest age of analysis. with ages t_0_ and t_n_ introduced into the same units;
F_Cl_: Environmental exposure factor for the initiation period, cm^3^·Ω/year.

Since the structure’s age is greater than 1 year, then applying any age factor and considering the value of the measured resistivity (ρ_e_) are not recommended without the additional factors of age or delay (Equation (6)). In this manner, service life can be calculated from the value of the resistivity of concrete and environmental and cement type factors. [64]. Although Equation (5) uses t_i_ and t_p_ estimates, the present investigation uses only t_i_.

t_i_ = (x^2^ × ρ_e_)/F_exp_ (6)

The F_exp_ values that can be used as a function of environmental aggressiveness are shown in Table 2 and are independent of the mechanical strength of the concrete [62].

## 3. Results and Discussion

### 3.1. Correlation of Variables Analyzed in Inspections

As described in Section 2.2, multiple correlations were performed, of which only comparisons showing proper correlations (i.e., R^2^ > 0.55 as we are using field data (uncontrolled environment)) are displayed. The analysis focuses on the global behavior of the data of the five ports as a whole to determine the behavior of concrete with different characteristics and elaborated under different conditions and to determine which index properties of the concrete are the most important from the point of view of durability. For all comparisons, an exponential type trend was proposed to analyze their exponent values and to estimate their degree of correlation. It is important to mention that the data presented are restricted only to concrete cores extractions permitted by the Port Authorities on each of the evaluated reinforced concrete piers. This is due to the commercial, historical and strategic importance of this infrastructure.

**Figure 3:**
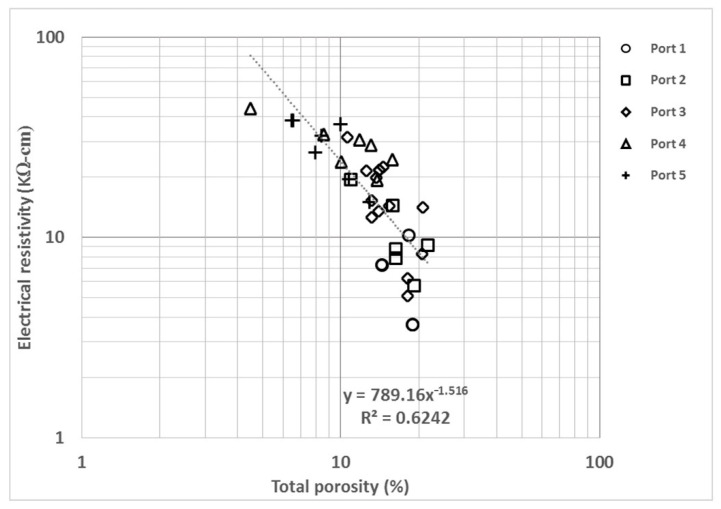
Electrical resistivity (ER) vs. total porosity (TP).

Figure 3 shows the comparison between total porosity (TP) and electrical resistivity (ER), with a regression coefficient of 0.62. An observed trend of the data with an exponent equal to −1.52 shows a strong correlation and inverse relation between the variables. TP is determined in the lab while ER can be measured in the field and the lab. This correlation allows the determination of a lab-based physical property in the field. Dispersion is observed in the data, and it is attributable to the coefficient of variation of the measurement methods (i.e., standard test methods for fluid ingress, such as bulk resistivity and bulk chloride diffusion). The measurement of ER shows a variation between 9.5% and 11%, as reported for the data from the same study, and up to 25% for the data from various studies [4]. Specifically, ER can be altered by aggregate rates [67,68], moisture content [69,70,71], cement content [72], type of cement [73] and chloride and sulfate content [70,74]. Nevertheless, the owners of the piers evaluated (the Port Authorities) did not have such information on the material properties (aggregates, cement, w/c ratio, etc.); thus, they are not included in the present investigation.

**Figure 4:**
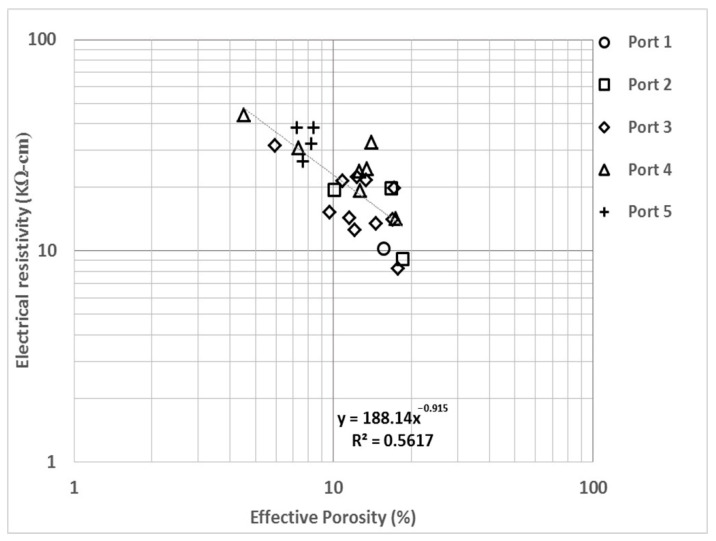
Electrical resistivity (ER) vs. effective porosity (EP).

In the empirical correlation of electrical resistivity (ER) with effective porosity (EP), Figure 4 shows an exponent value of −0.92 and regression coefficient of 0.56; these results denote a strong degree of correlation and an adequate regression value. Greater dispersion than the one shown in Figure 2 was observed, and the difference is attributable to the alterability of ER (as explained above). Moreover, they are samples of structures under field conditions, and their EP could be disturbed by macropores, curing and building quality. EP is a determining parameter for indicating the good behavior of concrete in the face of environmental degradation [17,75,76]. As ER and EP are correlated, the comparison is used to determine the reliability of the analyzed data and to detect any type of alteration in the measurements.

**Figure 5:**
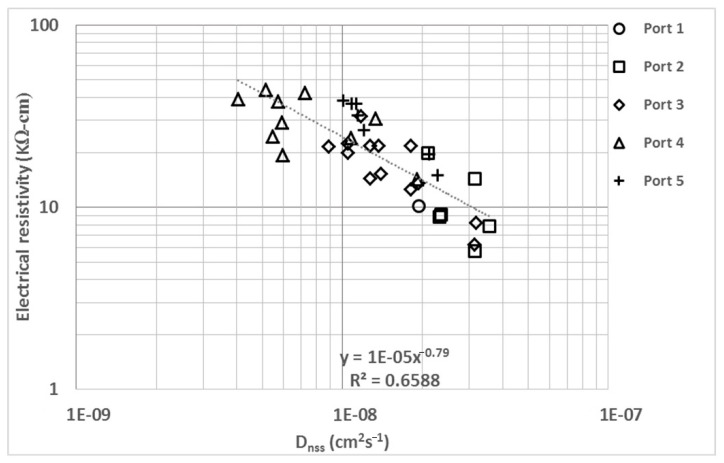
Electrical resistivity (ER) vs. non-steady state diffusion coefficient (D_nss_).

In Figure 5, ER is compared with D_nss_, which is necessary for the application of Fick’s life model, in addition to being determined by a high-uncertainty method (between 20% and 28%) [4]. This comparison facilitates the formulation of acceptable limits for D_nss_ in maritime infrastructure works; similar to ER, D_nss_ is affected by the type of cement [68,77] and moisture content [71]. This comparison has been reported with laboratory data by multiple researchers [38,68,69,71,78,79], and the results have shown behavior and regression similar to those reported in the current study.

**Figure 6:**
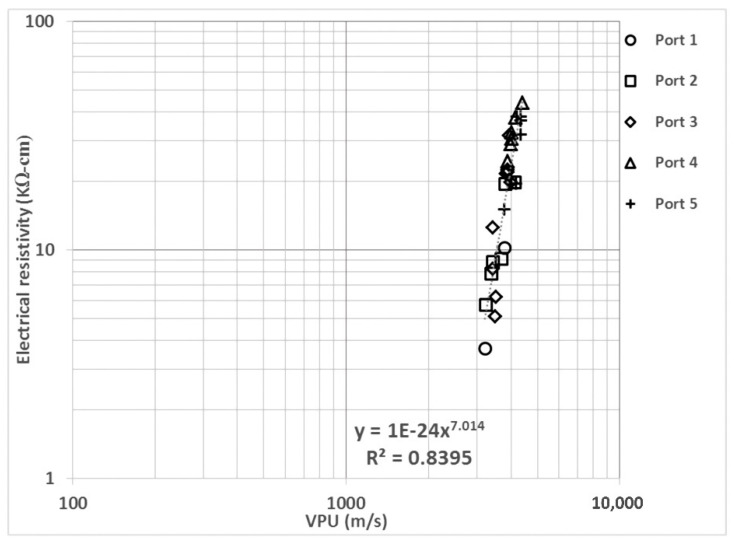
Electrical resistivity (ER) vs. ultrasonic pulse rate (UPV).

In Figure 6, ER and UPV are field (and lab) measurable, and the measures involve non-destructive tests. With this correlation and sensitivity analysis in life models, UPV acceptance limits for concrete in extremely aggressive environments could be reconsidered. UPV measurements are mainly affected by moisture content and cement type [68], showing differences in the values obtained from saturated vs. partially saturated specimens or cement type and compressive strength differences. In this case, we observe an excellent correlation, with an exponent value of 7.01 and a regression coefficient of 0.84.

**Figure 7:**
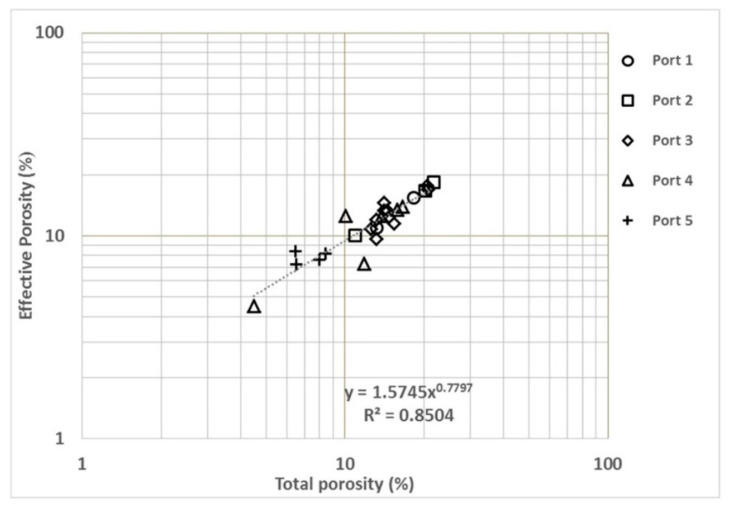
Effective porosity (EP) vs. total porosity (TP).

In this comparison (Figure 7), both variables are complementary lab measurements. EP is assumed to be only one part of TP. EP encompasses gel pores (from cement hydration) and capillary pores (from the expulsion of water from the mixture during concrete hardening) [80]. TP encompasses the previous two properties plus macropores, such as air bubbles and cavities emerging during the placement of concrete on the site. This comparison is useful for estimating both variables and determining any irregularities in any of the measurements. As shown in Figure 6, this empirical correlation shows an exponent value of around −0.78, which indicates a linear correlation trend. The regression coefficient is also high (R^2^ = 0.85).

**Figure 8:**
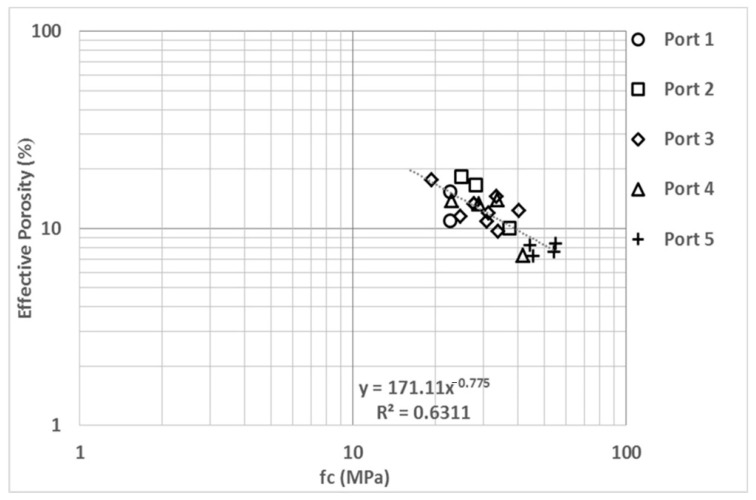
Effectiva porosity (EP) vs. compressive strength (fc).

The comparison between EP and mechanical strength of concrete (fc) (Figure 8) is useful for setting acceptance limits in terms of the minimum design resistance of the concrete used in seaports as this type of structure is usually designed to have a mechanical strength between 20 and 30 MPa. However, as observed in Figure 8, the durability requirements are not covered by these mechanical resistance values. Changes in quantity and type of cement can change the porosity of concrete; therefore, a high fc concrete tends to contain more cement and, consequently, has low porosity [4,5,72]. We observe a strong correlation with an exponent value of −0.78 and a regression coefficient of 0.63.

### 3.2. Estimation of t_i_ and Comparison of Models

**Figure 9:**
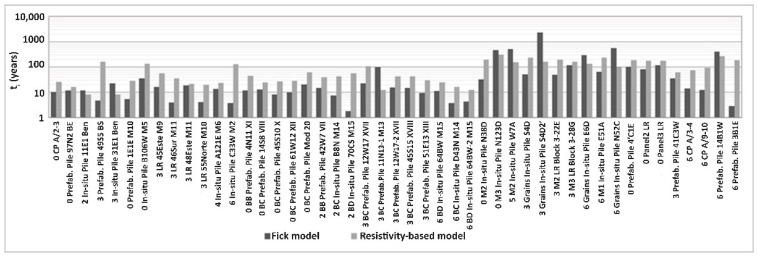
Comparison of models of t_i_ by element.

**Figure 10:**
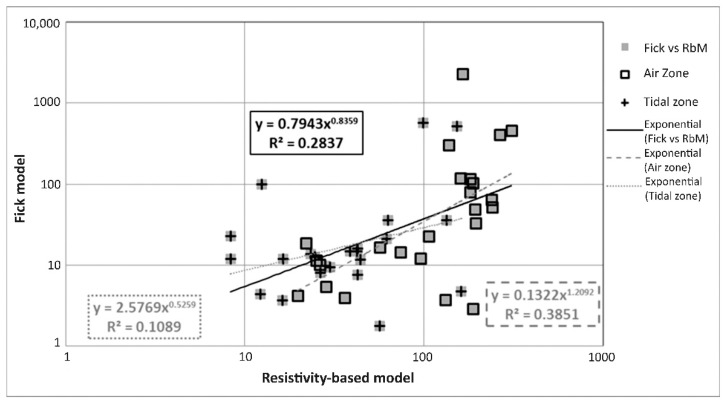
Fick’s model vs. Resistivity-based model (RbM) and exhibition zones.

Figure 9 and Figure 10 show the comparison and correlation of the analyzed Fick’s model and Resistivity-based model, with which the corrosion initiation time (t_i_) was determined for each of the 47 structural elements inspected in the five different ports. As shown in Figure 9, the comparison of the two models generates consistent results and does not present abrupt changes in the different elements. The differences in the results of the two models are due to the Resistivity-based model [62,63,66] considering environmental affectation factors and differentiating between tidal and air zones. Fick’s model only considers the gradient of Cl^−^ and the diffusivity of concrete [64,65]. The results of Fick’s law that are different from the rest correspond to the elements with low C_s_ (with values close to or below the limit concentration).

As the Resistivity-based model includes factors that depend on the type of environment to which the studied elements are exposed, both models should be compared by considering which area of each of the elements was analyzed (air zone or tidal zone). The correlation is presented in Figure 10. The elements with a relatively long shelf life are in the air zone where the concentrations of Cl^−^ and saturation levels were considerably low, thereby increasing diffusion time. Figure 10 also shows that no linearity exists in the behavior of the data because of the differences in the sensitivities of the models: Fick´s model presents higher variability when the Cl^−^ concentration gradient is low, especially when the surface concentration is very close to the limiting concentration, which is necessary with respect to corrosion initiation. In these cases, the concentration gradient can be equal to or slightly lower than the limiting concentration, which drastically raises the value of t_i_. This is in accordance with Poulsen and Mejlbro [64] (pp. 3–4) since they expose that the change of chloride concentration per unit of time is equal to the change of flux per unit of length, and in turn flux is proportional to the concentration gradient normal to the section of the exposed element concerning Resistivity-based model where ER changes produce proportional changes on t_i_.

The regressions presented in Figure 10 are low because of data spread. Several values behave similarly to outliers. According to the analysis of the comparison graphs of the variables described above, we determine that such outliers are due to alterations in ER and/or diffusivity data (both data are experimental and the basis for feeding the analyzed models). Although the regression coefficient is weak for all trends, the exponent values denote a strong correlation for the air zone the total trend (1.21 and 0.84, respectively).

**Figure 11:**
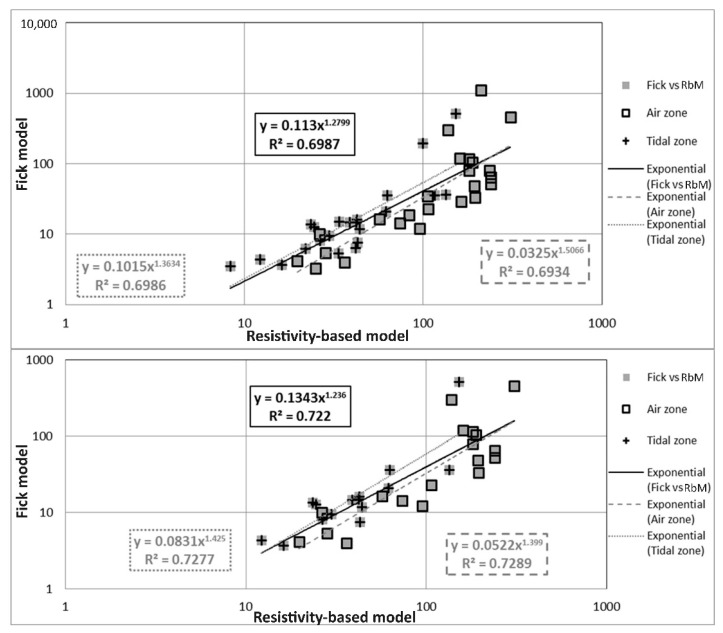
Fick’s model vs. Resistivity-based model (RbM) and exhibition zones, with correction of outliers (up) and without outliers (down).

Using the correlation equations shown in Section 3.1, the deletion of outliers and/or correction of atypical data were expected. In order to determine if one of the analyzed data performs atypical, R^2^ estimates were used as an acceptance parameter. For example, the correlation coefficient R^2^ for EP vs. TP (Figure 7) was 0.85, then a 15% acceptance value was considered for this empirical correlation. Therefore, any data with a lower or higher difference than 15% were considered as atypical; for example, if any of the data of this graph (Figure 7) were out of this 15% limit, the data were eliminated or corrected using the correlation equation determined in Section 3.1. Figure 11 shows the comparison of the models by correcting (using data cleaning/inference and data quality/range constraints) and deleting the outliers to achieve significant improvements in correlation.

### 3.3. Comparison of t_i_ with Studied Variables

**Figure 12:**
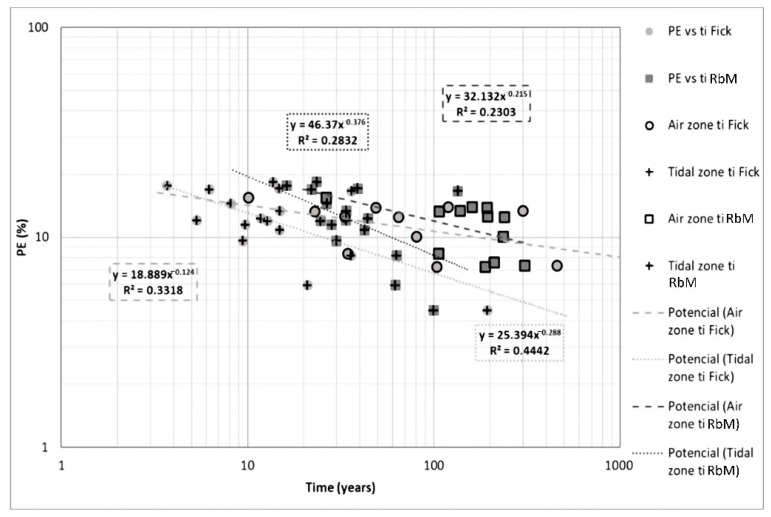
EP vs. t_i_ in both zones and models.

**Figure 13:**
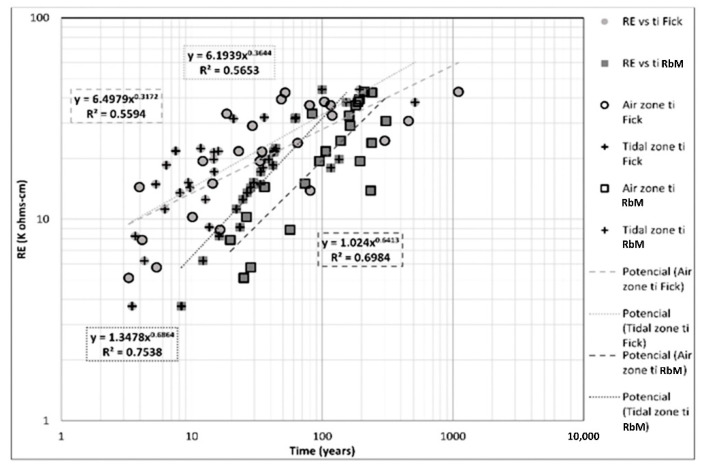
ER vs. t_i_ in both zones and models.

**Figure 14:**
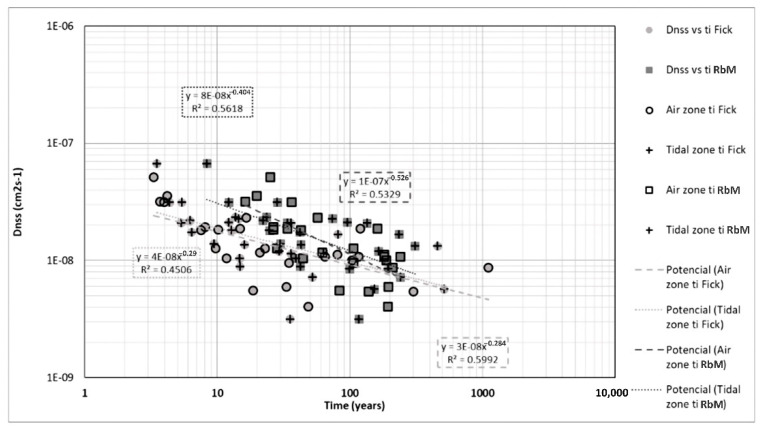
D_nss_ vs. t_i_ in both zones and models.

In order to determine whether both models were sensitive to any of the previously analyzed variables, we compared the initiation times of both models and variables. Figure 12, Figure 13 and Figure 14 show the results of the comparisons with improved correlations. For comparisons between t_i_ and the physical–mechanical variables of concrete, we use only the data including the correction of outliers (only the corrected data are used because they allow us to keep as much data as possible for the comparison of t_i_ with the index properties).

Figure 12 compares t_i_ with EP (mentioned above). EP is a defining parameter for determining the durability of concrete. Figure 12 shows a relatively high regression coefficient for the tidal zone of Fick’s model, but it does not present any adequate correlation for UNE’s model. If a good correlation is present in the comparison between ER and EP, a good correlation between the UNE’s model and the EP would be expected.

Figure 13 presents the comparison between ER and initiation time. Resistivity-based model shows a clearer trend and a higher correlation than Fick’s model. Meanwhile, Fick’s model shows a less clear trend than Resistivity-based model, but regression coefficients of 0.56 and 0.57, respectively, indicate a possible correlation with this variable in tidal and aerial zones.

In the case of the diffusion coefficient, considerably small regressions were observed relative to t_i_ (Figure 14), showing the highest values in both zones using Resistivity-based model and air zone in Fick´s; the tidal zone in Fick’s model barely achieves a 0.45 regression coefficient. This comparison shows extensive data dispersion, especially for Fick’s model. Although correlations are weak, they show that the coefficient of diffusion of chlorides is an important variable in estimating the life of concrete, as documented by Zhang and Zoubir [81]. The model presented by Zhang and Zoubir is a diffusion-based corrosion initiation model for reinforced concrete structures in chloride-laden environments. They used four parameters to determine the sensitivity of the time to corrosion initiation: chloride diffusivity in concrete, chloride threshold level in reinforcing steel, concrete cover and surface chloride concentration. For conventional carbon steel, the time to corrosion initiation is most sensitive to concrete cover.

## 4. Conclusions

This research highlights the feasibility of implementing comparisons of the different index properties of concrete to determine the reliability of different data obtained during detailed inspections performed to marine naturally exposed reinforced concrete piers by altered data elimination obtained from random bias (human or complexity of the inspection errors). The results of these data are necessary for the estimation of concrete service life and the identification of preventive measures against environmental degradation. Thus, we identified the useful variables or index properties as saturated ER, percent total void content, capillary (or effective) porosity, non-steady state chloride diffusion coefficient, ultrasonic pulse velocity and compressive strength of concrete. By focusing on these concrete properties, an adequate design of concrete structures exposed to corrosive environments can be achieved.

During inspections for the durability of concrete structures, we can determine which data can be considered atypical and be properly treated within the life prediction models by using the correlations of variables for the index properties. Figure 10 and Figure 11 show that with correct identification and handling of atypical data, a significant improvement in the correlation of the variables can be achieved. This study achieves an improvement of R^2^ in the comparison between the t_i_ models analyzed (Figure 10). An increase in R^2^ from 0.28 to 0.70 and 0.72 was achieved by, respectively, using correction and elimination of the general trend with the correction and deletion of outlier data; for the air zone, an improvement in R^2^ from 0.39 to 0.69 and 0.73 was respectively, achieved while for the tidal zone, an improvement in R^2^ from 0.11 to 0.70 and 0.73 was, respectively, realized.

Regarding the comparison of these variables with the initial time of corrosion, adequate correlations were obtained only for EP, chloride diffusion coefficient and ER. Although all the comparisons show great variability even with the handling of outlier data, the variations may be due to the quality control of each construction and design method, with cement’s quantity and quality varying depending on the design method for concrete mixtures. Another alternative may be the type and quality of aggregates used in each port, as several authors have reported that the characteristics of aggregates can alter the physical properties of concrete [17,42,75,82].

ER is verified as a variable with strong correlations because it relates to four of the five variables analyzed at the level of the index properties of concrete and at the level of calculation of corrosion initiation time. Thus, we conclude that the most decisive property for good concrete performance is ER, which can be used in design and construction control and the inspection stage.

## Figures and Tables

**Table 1:** Naming and location of ports.

Port	Coast	Tropic
Port 1	Pacific	Above the Tropic of Cancer
Port 2	Gulf of Mexico	Below the Tropic of Cancer
Port 3	Pacific	Below the Tropic of Cancer
Port 4	Pacific	Below the Tropic of Cancer
Port 5	Pacific	Below the Tropic of Cancer

**Table 2:** Environmental exposure factor (F_exp_) values as a function of the type of environmental exposure.

Exposition Type	Fexp (cm^3^·Ω/year)
Corrosion of non-chloride origin (high humidity).	-
Corrosion of non-chloride origin (mid-range humidity).	-
Marine exposure in the air zone. Chloride corrosion in elements of marine structures, above the high tide level (>500 m from the coast).	5000
Marine exposure in the air zone. Chloride corrosion in elements of marine structures, above the high tide level (<500 m from the coast).	10,000
Marine exposure in the submerged zone. Chloride corrosion in elements of permanently submerged marine structures, below the minimum low tide level.	17,000
Marine exposure in tidal and splash zone. Chloride corrosion on elements of marine structures located in the splash zones or the tidal race zone.	25,000

## Data Availability

Not applicable.

## References

[1] Tang S.W., Yao Y., Andrade C., Li Z.J. (2015). Recent durability studies on concrete structure. Cem. Concr. Res..

[2] Alexander M., Beushausen H. (2019). Durability, service life prediction, and modelling for reinforced concrete structures–review and critique. Cem. Concr. Res..

[3] Hooton R.D. (2019). Future directions for design, specification, testing, and construction of durable concrete structures. Cem. Concr. Res..

[4] ACI (2004). Building Code Requirements for Structural Concrete and Commentary.

[5] CEN (2002). EuroCode2: Design of Concrete Structures.

[6] Ministry of Housing and Urban-Rural Development of the People’s Republic of China (2008). Code for Durability Design of Concrete Structures.

[7] Tuutti K. (1982). Corrosion of Steel in Concrete, CBI Research Report 4:82.

[8] Zhang J., Lounis Z. (2009). Nonlinear relationships between parameters of simplified diffusion-based model for service life design of concrete structures exposed to chlorides. Cem. Concr. Compos..

[9] Stratfull R.F. (1956). The corrosion of steel in a reinforced concrete bridge. Corrosion.

[10] Gouda V.K. (1970). Corrosion and corrosion inhibition of reinforcing steel. Br. Corr. J..

[11] Rosenberg A., Hansson C.M., Andrade C., Skalny J. (1989). Mechanisms of corrosion of steel in concrete. Materials Science of Concrete.

[12] Cady P.D., Weyers R.E. (1983). Chloride penetration and deterioration of concrete bridge decks. Cem. Concr. Aggreg..

[13] Sarja A., Vesikari E. (1996). Durability Design of Concrete Structures: Report of Rilem Technical Committee 130-Csl.

[14] Kropp J. (1995). Chlorides in concrete. Performance Criteria for Concrete Durability. RILEM Report 12.

[15] Hooton R.D. (2016). Achieving concrete durability in chloride exposures. Key Eng. Mater..

[16] Hooton R.D., Colombo M., di Prisco M. Improved Sustainability by Design for Concrete Durability. Proceedings of the 8th International Conference on Concrete under Severe Conditions-Environment & Loading.

[17] Neville A.M. (2011). Properties of Concrete.

[18] Amriou A., Bencheikh M. (2017). New experimental method for evaluating the water permeability of concrete by a lateral flow procedure on a hollow cylindrical test piece. Constr. Build. Mater..

[19] Alarcon-Ruiz L., Brocato M., Dal Pont S., Feraille A. (2010). Size effect in concrete intrinsic permeability measurements. Transp. Porous Media.

[20] Hearn N. (1998). Self-sealing, autogenous healing and continued hydration: What is the difference?. Mater. Struct..

[21] Loosveldt H., Lafhaj Z., Skoczylas F. (2002). Experimental study of gas and liquid permeability of a mortar. Cem. Concr. Res..

[22] Charron J.P., Denarié E., Brühwiler E. (2008). Transport properties of water and glycol in an ultra-high performance fiber reinforced concrete (UHPFRC) under high tensile deformation. Cem. Concr. Res..

[23] Tam C.M., Tam V.W.Y., Ng K.M. (2012). Assessing drying shrinkage and water permeability of reactive powder concrete produced in Hong Kong. Constr. Build. Mater..

[24] Powers T.C. (1958). Structure and physical properties of hardened Portland cement paste. J. Am. Ceram. Soc..

[25] Lane D.S., Detwile R.L., Hooton R.D. (2010). Testing transport properties in concrete. Concr. Int..

[26] Christensen B.J., Mason T.O., Jennings H.M. (1996). Comparison of measured and calculated permeabilities for hardened cement pastes. Cem. Concr. Res..

[27] Das B.B., Kondraivendhan B. (2012). Implication of pore size distribution parameters on compressive strength, permeability and hydraulic diffusivity of concrete. Constr. Build. Mater..

[28] Goto S., Roy D.M. (1981). The effect of W-C ratio and curing temperature on the permeability of hardened cement paste. Cem. Concr. Res..

[29] Nyame B.K., Illston J.M. (1981). Relationships between permeability and pore structure of hardened cement paste. Mag. Concr. Res..

[30] Dhir R.K., Hewlett P.C., Chan Y.N. (1989). Near surface characteristics of concrete: Intrinsic permeability. Mag. Concr. Res..

[31] Hooton R.D. (2008). Bridging the gap between research and standards. Cem. Concr. Res..

[32] Hwan-Oh B., Jang S.Y. (2007). Effects of material and environmental parameters on chloride penetration profiles in concrete structures. Cem. Concr. Res..

[33] Mehta P.K. (1986). Concrete: Structure, Properties and Materials.

[34] Khelidj A., Loukili A., Bastian G. (1998). Experimental study of the hydro-chemical coupling inside maturing concretes: Effect on various types of shrinkage. Mater. Struct..

[35] Nielsen E.P., Geiker M.R. (2003). Chloride diffusion in partially saturated cementitious material. Cem. Concr. Res..

[36] Zhiwu Y., Chen Y., Liu P., Wang W. (2015). Accelerated simulation of chloride ingress into concrete under drying-wetting alternation condition chloride environment. Constr. Build. Mater..

[37] Chunhua L., Yuan G., Zhaowei C., Ronggui L. (2015). Experimental analysis of chloride penetration into concrete subjected to drying-wetting cycles. J. Mater. Civil. Eng..

[38] Polder R.B., Willy H.A. (2002). Characterization of chloride transport and reinforcement corrosion in concrete under cyclic wetting and drying by electrical resistivity. Cem. Concr. Compos..

[39] Kuosa H., Ferreira R.M., Holt E., Leivo M., Vesikari E. (2014). Effect of coupled deterioration by freeze–thaw, carbonation and chlorides on concrete service life. Cem. Concr. Compos..

[40] Baroghel-Bouny V., Wang X., Thiery M., Saillio M., Barberon F. (2012). Prediction of chloride binding isotherms of cementitious materials by analytical model or numerical inverse analysis. Cem. Concr. Res..

[41] Yuan Q., Shi C., De Schutter G., Audenaert K., Deng D. (2009). Chloride binding of cement-based materials subjected to external chloride environment—A review. Constr. Build. Mater..

[42] Neville A. (1995). Chloride attack of reinforced concrete: An overview. Mater. Struct..

[43] Suryavanshi A.K., Scantlebury J.D., Lyon S.B. (1998). Corrosion of reinforcement steel embedded in high water–cement ratio concrete contaminated with chloride. Cem. Concr. Compos..

[44] Reddy B., Glass G.K., Lim P.J., Buenfeld N.R. (2002). On the corrosion risk presented by chloride bound in concrete. Cem. Concr. Compos..

[45] McGrath P.F. (1996). Development of Test Methods for Predicting Chloride Penetration into High Performance Concrete. Ph.D. Thesis.

[46] Martin-Perez B., Zibara H., Hooton R.D., Thomas M.D.A. (2000). A study of the effect of chloride binding on service life predictions. Cem. Concr. Res..

[47] Collepardi M., Marcialis A., Turriziani R. (1972). Penetration of chloride ions into cement pastes and concretes. J. Am. Ceram. Soc..

[48] General Coordination of Ports and Merchant Marine, Directorate-General for Ports, Directorate of Port Development (2008). National Port Development Program 2007–2030.

[49] Del Valle-Moreno A., Fabela-Gallegos M.J., Hernandez-Jimenez J.R., Vazquez-Vega D., Torres-Acosta A.A., Teran-Guillen J., Martinez-Madrid M. (2011). Determination of the corrosion status and load capacity of the docks of the port of Guaymas, Technical Publication. IMT.

[50] Uller L., Trocónis O., Alanis I., Helene P., Mejías R., O’Reilly V., Andrade C., Carpio J.J., Díaz I., Salta M. (1998). DURAR Thematic network XV.B, Durability of the armor. Manual of Inspection, Evaluation and Diagnosis of Corrosion in Reinforced Concrete Structures.

[51] Frangopol B.D.M., Kong J.S., Gharaibeh E.S. (2001). Reliability-based life-cycle management of highway bridges. J. Comput. Civ. Eng..

[52] Biondini F., Frangopol D.M. (2016). Life-cycle performance of structural systems under uncertainty. J. Struct. Eng..

[53] Akcamete A., Akinci B., Garrett J.H. Potential utilization of building information models for planning maintenance activities. Proceedings of the ICCCBE.

[54] Barone G., Frangopol D.M. (2014). Life-cycle maintenance of deteriorating structures by multi-objective optimization involving reliability, risk, availability, hazard and cost. Struct. Saf..

[55] Lepech M.D., Geiker M., Michel A., Stang H. Probabilistic design and management of sustainable concrete infrastructure using multi-physics service life models. Proceedings of the 1st International Conference on Grand Challenges in Construction Materials.

[56] Becerik-Gerber B., Jazizadeh F., Li N., Calis G. (2011). Application areas and data requirements for BIM-enabled facilities management. J. Constr. Eng. Manag..

[57] Li C.Q. (2003). Life-cycle modeling of corrosion-affected concrete structures: Propagation. J. Struct. Eng..

[58] Li Q., Ye X. (2018). Surface deterioration analysis for probabilistic durability design of RC structures in marine environment. Struct. Saf..

[59] Bazant Z.P. (1979). Physical model for steel corrosion in concrete sea structures-theory. J. Struct. Div..

[60] Bamforth P.B. (1999). The derivation of input data for modelling chloride ingress from eightyear UK coastal exposure trials. Mag. Concr. Res..

[61] Li C.Q. (2000). Corrosion initiation of reinforcing steel in concrete under natural salt spray and service loading—results and analysis. Mater. J..

[62] Andrade C. (2018). Design and evaluation of service life through concrete electrical resistivity. Rev. ALCONPAT.

[63] Andrade C., Weiss J., Kovler K., Marchand J., Mindess S. (2004). Calculation of initiation and propagation periods of service-life of reinforcements by using the electrical resistivity. Proceedings of the International Symposium on Advances in Concrete Through Science and Engineering, RILEM Symposium.

[64] Poulsen E., Mejlbro L. (2006). Modern Concrete Technology T&F. Difusion of Chloride in Concrete, Theory and Application.

[65] Mangat P.S., Molloy B.T. (1994). Prediction of long term chloride concentration in concrete. Mater. Struct..

[66] Andrade C., Rebolledo N., Tavares F., Pérez R., Baz M. (2016). Concrete durability of the new Panama Canal: Background and aspects of testing. Mar. Concr. Struct..

[67] Tsung-Chin H., Van Kien N., Yu-Min S., Yuan-Rong C., Pei-Ju C. (2017). Effects of coarse aggregates on the electrical resistivity of Portland cement concrete. Constr. Build. Mater..

[68] Kurda R., de Brito J., Silvestre J.D. (2019). Water absorption and electrical resistivity of concrete with recycled concrete aggregates and fly ash. Cem. Concr. Compos..

[69] Climent M.A., de Vera G., López J.F., Viqueira E., Andrade C. (2002). A test method for measuring chloride diffusion coefficients through nonsaturated concrete Part I. The instantaneous plane source diffusion case. Cem. Concr. Res..

[70] Saleem M., Shameen M., Hussain S.E., Maslehuddin M. (1996). Effect of moisture, chloride and sulphate contamination on the electrical resistivity Portland cement concrete. Constr. Build. Mater..

[71] De Vera G., Climent M.A., Viqueira E., Antón C., Andrade C. (2007). A test method for measuring chloride diffusion coefficients through partially saturated concrete. Part II: The instantaneous plane source diffusion case with chloride binding consideration. Cem. Concr. Res..

[72] Linares-Alemparte P., Andrade C., Baza D. (2019). Porosity and electrical resistivity-based empirical calculation of the oxygen diffusion coefficient in concrete. Constr. Build. Mater..

[73] Medeiros-Junior R.A., Lima M.G. (2016). Electrical resistivity of unsaturated concrete using different types of cement. Constr. Build. Mater..

[74] Fares M., Villain G., Bonnet S., Palma Lopes S., Thauvin B., Thiery M. (2018). Determining chloride content profiles in concrete using an electrical resistivity tomography device. Cem. Concr. Compos..

[75] Newman J.C., Choo B.S. (2003). Advanced Concrete Technology Constituent Materials.

[76] Chang H., Mu S., Xie D., Wang P. (2017). Influence of pore structure and moisture distribution on chloride ‘‘maximum phenomenon” in surface layer of specimens exposed to cyclic drying-wetting condition. Constr. Build. Mater..

[77] Franco-Luján V.A., Maldonado-García M.A., Mendoza-Rangel J.M., Montes-García P. (2019). Chloride-induced reinforcing steel corrosion in ternary concretes containing fly ash and untreated sugarcane bagasse ash. Constr. Build. Mater..

[78] Andrade C., d’Andrea R., Rebolledo N. (2014). Chloride ion penetration in concrete: The reaction factor in the electrical resistivity model. Cem. Concr. Compos..

[79] Torres-Acosta A.A., Presuel-Moreno F., Andrade C. (2019). Electrical resistivity as durability index for concrete structures. ACI Mater. J..

[80] Neville A.M., Brooks J.J. (2010). Concrete Tecnology.

[81] Zhang J., Zoubir L. (2006). Sensitivity analysis of simplified diffusion-based corrosion initiation model of concrete structures exposed to chlorides. Cem. Concr. Res..

[82] Kosmatka H.S. (2004). Diseño y Control de Mezclas de Concreto.

